# Exploring the Role of Pheromones and CRISPR/Cas9 in the Behavioral and Olfactory Mechanisms of *Spodoptera frugiperda*

**DOI:** 10.3390/insects17010035

**Published:** 2025-12-25

**Authors:** Yu Wang, Chen Zhang, Mei-Jun Li, Asim Iqbal, Kanwer Shahzad Ahmed, Atif Idrees, Habiba, Bai-Ming Yang, Long Jiang

**Affiliations:** 1 Agricultural College, Jilin Agricultural Science and Technology College, Jilin 132101, China; wangyu@jlnku.edu.cn (Y.W.); zhangchenjl@163.com (C.Z.); 2 Institute of Peanut Research, Jilin Academy of Agricultural Science/Jilin Key Laboratory of Agricultural Microbiology/Key Laboratory of Integrated Pest Management on Crops in Northeast China, Ministry of Agriculture and Rural Areas, Gongzhuling 136100, China; 15134658066@163.com; 3 Imdaad: Integrated Facilities Management Company, Street Number 1100, South Zone Jebel Ali, Dubai P.O. Box 18220, United Arab Emirates; asim_iqbal990@yahoo.com; 4 Biological Research & Resource Center, Mastermind Scientific Consultants (SMC-Private) Limited, Sargodha 40100, Punjab, Pakistan; rajput.shahzad18@gmail.com; 5 Guizhou Provincial Key Laboratory for Agricultural Pest Management of the Mountainous Region, Scientific Observing and Experimental Station of Crop Pest in Guiyang, Ministry of Agriculture and Rural Affairs, Institute of Entomology, Guizhou University, Guiyang 550025, China; atifentomologist@gmail.com; 6 Lehman College, City University of New York, S-3401/3402, 250 Bedford Park Blvd West, Bronx, NY 10468, USA; fnu.habiba79@login.cuny.edu

**Keywords:** CRISPR/Cas system, odorant receptors, olfactory system, *Orco* knockout, sex pheromone, *Spodoptera frugiperda*

## Abstract

Globally, *Spodoptera frugiperda* is a major threat to many important crops, including maize, rice, and cotton, causing significant economic damage. To control this invasive pest, environmentally friendly pest control techniques, including pheromone detection and identification of potential molecular targets to disrupt *S. frugiperda* mating communication, are needed. Female moths biosynthesize pheromones and emit them from the pheromone gland, which significantly depends on the intrinsic factors of the moth. Male *S. frugiperda* have a sophisticated olfactory circuit on their antennae that recognizes pheromone blends via olfactory receptor neurons (ORNs). With its potential to significantly modify the insect genome, CRISPR/Cas9 offers a revolutionary strategy to control this insect pest. The impairing physiological behaviors and disrupting the *S. frugiperda* volatile-sensing mechanism are the main potential applications of CRISPR/Ca9 explored in this review. Furthermore, the release of mutant *S. frugiperda* for their long-term persistence must be integral to the adoption of this technology. Looking forward, CRISPR/Cas9-based gene drive systems have the potential to synergistically target pheromone signaling pathways in *S. frugiperda* by disrupting pheromone receptors and key biosynthesis genes, thereby effectively blocking intraspecific communication and reproductive success. In conclusion, CRISPR/Cas9 provides an environmentally friendly and revolutionary platform for precise, targeted pest management in *S. frugiperda*.

## Simple Summary

*Spodoptera frugiperda*, a significant global agricultural pest, poses a substantial threat to maize and other crops, resulting in considerable yield losses. To control this pest, CRISPR/Cas9 is considered a state-of-the-art genetic control strategy, as it significantly disrupts *S. frugiperda* mating communication and volatile sensing mechanisms. After overcoming the challenges associated with CRISPR/Cas9 application, it remains a powerful, new, environmentally friendly, and revolutionary platform for precise, targeted pest management in *S. frugiperda*.

## 1. Introduction

Genome editing, commonly known as gene editing, involves inserting, deleting, labeling, or reordering genetic material to produce a desired trait through genetic manipulation [1,2]. The Clustered Regularly Interspaced Short Palindromic Repeats (CRISPR)/CRISPR-associated protein 9 (Cas9) originates from the archaeal and bacterial immune systems and is the most advanced and superior genome editing technology [3,4]. The CRISPR/Cas9 system consists of the Cas9 protein, which cleaves double-stranded DNA (dsDNA) to facilitate genome modification, and a chimeric 20 bp single-guide RNA (sgRNA) that instructs Cas9 to target the desired sequence [5]. The mechanism of CRISPR/Cas9 genome editing comprises three steps: recognition, cleavage, and repair [6]. Over the past ten years, the insect-resistant plants and various insects have been modified using CRISPR-Cas gene editing for agricultural insect pest management [7]. This gene-editing tool has altered the genomes of many insects, affecting biological and physiological traits such as pigmentation, reproduction, development, insecticide resistance, metabolism, olfaction, and body segmentation [8]. A previous study conducted by Sun et al. [9] also highlighted the application of CRISPR-Cas9 in insects and non-insect arthropods. Additionally, this technology is pivotal in advancing economic insect breeding by being applied to *Bombyx mori* Linnaeus, 1758, thereby enhancing disease resistance and improving silk quality [10]. In addition, CRISPR-Cas9 can disrupt pheromone recognition in insects by targeting and altering genes involved in pheromone recognition, which is recognized as the most efficient and promising, eco-friendly alternative for the sustainable management of major insect pests worldwide [11,12].

Globally, *Spodoptera frugiperda* is an invasive and one of the major pest insects in diverse crop plants, including maize, rice, and cotton [13], that causes significant economic damage [14]. It is native to the tropical and subtropical regions of the Americas, where it accounted for about 36% of the annual loss of maize production in sub-Saharan Africa, reducing maize production by 0.67 million tons between 2017 and 2019 [15,16]. The extensive and indiscriminate use of insecticides to control this insect pest has led to the evolution of insecticide resistance and also caused ecotoxicological effects on non-target insects [17,18]. Therefore, manipulating the olfactory-guided behaviors of insects provides new avenues for controlling insect pests [19]. Currently, disrupting pheromone recognition gene in *S. frugiperda* using CRISPR-Cas9-mediated genome editing is the most promising and effective pest management technique [11,12]. In *S. frugiperda*, the 8th and 9th intersegmental membranes produce sex pheromones, which the insect uses to find mates, identify species, and select mates [20]. Furthermore, insect pest physiological parameters, such as age and mating status, can affect pheromone production, providing valuable information for pest management [21].

A pheromone communication system is where male insects recognize the pheromone signals to identify a potential mate, ensuring reproductive success [22]. The (Z)-9-tetradecenyl acetate (Z9–14:OAc), a crucial sexual communication signal produced by *S. frugiperda* females [23,24,25,26], can be detected by males even at minute concentrations [27]. Olfactory receptor neurons (ORNs) are dispersed across several appendages on the antennae and express obligate odorant receptor co-receptors (*Orco*) [28,29] coupled with a “tuning” odorant receptor (OR) to form heteromeric, odor-gated ion channels in the membranes of these neurons to facilitate the chemosensory process [30,31,32]. These results provide a solid foundation for understanding advances in the molecular mechanisms of *S. frugiperda* sex pheromone biosynthesis and identify new targets for developing novel pest control methods that disrupt sexual communication. Currently, the olfactory-based pest control strategies are considered most promising and effective, in which various behavioral processes were impaired by mutagenesis of *Orco* across multiple insect pests such as World screwworm, *Cochliomyia hominivorax*, Coquerel [33], cotton bollworm, *Helicoverpa armigera* Hubner [34] hawkmoth, *Manduca sexta* Linnaeus [35]. However, research on *Orco* mutagenesis in *S. frugiperda* remains limited; therefore, this review focuses on the biosynthesis, release, and recognition of sex pheromones in *S. frugiperda*, elucidating the role of the olfactory system in mediating critical behaviors such as mating, foraging, and oviposition. Furthermore, we have evaluated the potential of CRISPR/Cas9-mediated gene editing, particularly *Orco* disruption, in functionally characterizing pheromone receptor pathways and altering behavioral response.

## 2. Pheromone Dynamics and Recognition in *S. frugiperda*

### 2.1. Biosynthesis of Sexual Pheromone in S. frugiperda

**Figure 1:**
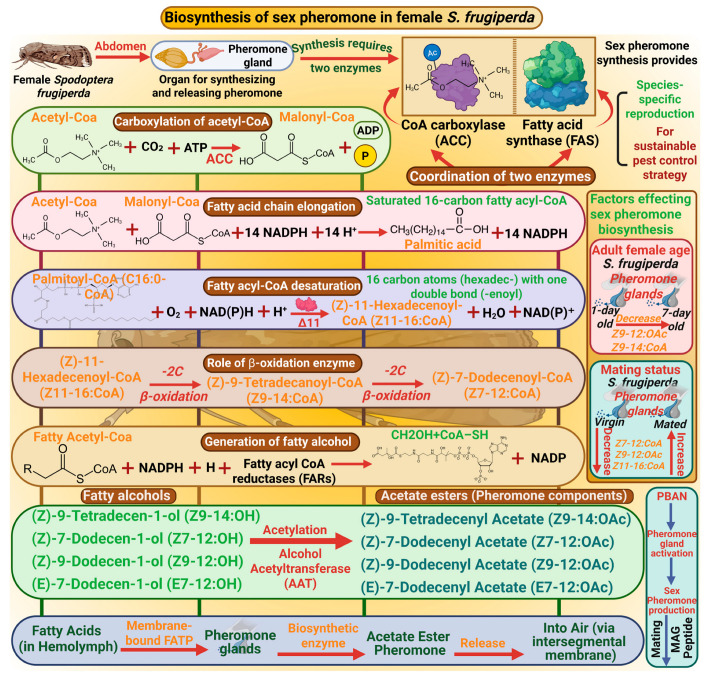
Biosynthesis of the sex pheromone in female *Spodoptera frugiperda* involves key enzymes, such as CoA carboxylase and fatty acid synthase, which coordinate fatty acid synthesis. These fatty acids are desaturated and oxidized, then converted into fatty alcohols and acetate esters, which are the main pheromone components. This process is regulated by PBAN (pheromone biosynthesis-activating neuropeptide), which activates the pheromone production and attracts males for mating. “Figure created using Biorender.com”.

The biosynthesis of sexual pheromones in *S. frugiperda* is central to understanding species-specific reproduction and offers a vital basis for sustainable pest control strategies. In *S. frugiperda*, pheromone glands are present in the abdomen, essential organs for synthesizing and releasing pheromones [36]. The synthesis of pheromones requires the coordination of different types of enzymes, including acetyl-CoA carboxylase (ACC) and fatty acid synthase (FAS). ACC catalyzes the carboxylation of acetyl-CoA to malonyl-CoA [37]. Then FAS catalyzes the condensation of malonyl CoA units with NADPH as a reducing agent to elongate the fatty acid chain and produces the palmitic acid (C16:0-CoA), a saturated 16-carbon fatty acyl-CoA [38]. Thus, fatty acyl-CoA desaturation occurred by enzyme Δ11 desaturase to generate unsaturated fatty acids such as (Z)-11-hexadecenoyl-CoA (Z11–16-CoA) by introducing a double bond at the 11th carbon of palmitic acid. After this desaturation, beta-oxidation enzymes shorten the fatty acyl-CoA chain by removing two carbon units, producing intermediate molecules such as Z9–14:OAc and (Z)-7-dodecenyl acetate (Z7–12:CoA). These precursors are then reduced by fatty acyl CoA reductases (FARs) to generate fatty alcohols, which are important intermediates in pheromone formation. Furthermore, Alcohol Acetyltransferase (AAT) enzyme catalyzes acetylation to produce acetate esters such as (Z)-9-tetradecenyl acetate (Z9–14:OAc), (Z)-7-dodecenyl acetate (Z7–12:OAc), (Z)-9-dodecenyl acetate (Z9–12:OAc), and (E)-7-dodecenyl acetate (E7–12:OAc), which are ultimate pheromone components [39]. A membrane-bound fatty acid transport protein carries fatty acids through hemolymph to the pheromone gland, synthesizing pheromones [24]. The female moth emits pheromone from the pheromone gland which is located at the intersegmental membrane between the 8th and 9th abdominal segments [40] (Figure 1).

### 2.2. Factors Affecting Sex Pheromone Release from Female S. frugiperda

Age and mating status are the most promising factors influencing the release of sex pheromones from female *S. frugiperda*, and these factors are explained in detail.

#### 2.2.1. Adult Female Age

Insect aging is the crucial factor that influences the release of pheromone. In this context, Akter et al. [41] investigated the pheromone glands of females *S. frugiperda* between the 4th and 6th hours of the scotophase. After extracting glands of varying female ages, it was found that the release of volatile compounds such as Z9–12:OAc and Z9–14:CoA was higher in one-day-old females compared with four-day and seven-day-old females (*p* < 0.001 and *p* < 0.01, respectively). Therefore, aging has a negative impact on the pheromone release from *S. frugiperda* females (Figure 1). Furthermore, while collecting headspace volatiles from female moths of various ages also indicated that younger females released and produced higher amounts of compounds than older ones [41]. Similarly, Domínguez et al. [21] reported that pheromone release from glands is significantly reduced as the female age increases. Because pheromone production is governed by the endocrine system, aging in insects negatively influences the endocrine system by decreasing juvenile hormone production from the corpora allata, reducing the sensitivity of both endocrine tissues and pheromone receptor cells, diminishing ovarian activity and weekend neural regulation of endocrine glands, and ultimately reducing pheromone production [42,43]. Consequently, one-day-old females release pheromones with enhanced mate-attraction potential and achieve higher mating success rates than older individuals. Therefore, these findings suggest that younger females produce more pheromones to boost reproductive fitness and mating chances.

#### 2.2.2. Mating Status

Mating dramatically alters the pheromone release, often reducing or stopping sexual activity in females to avoid further mate attention and focused reproduction. Therefore, Akter et al. [41] revealed that mated females had retained significantly higher concentrations of headspace volatiles, including Z7–12:CoA, Z9–12:OAc, and Z11–16-CoA, in their glands compared to virgin females. The production of these volatile sex pheromones is regulated by the pheromone biosynthesis-activating neuropeptide (PBAN), which is predominantly produced by neurosecretory cells of the subesophageal ganglion and transported to the corpora cardiaca before its release into the hemolymph [44]. Mating triggers the transfer of a sex peptide, a pheromone depressant peptide, from the male’s accessory gland (MAG) to the female genital tract, ultimately triggering juvenile hormone production in the corpus allatum in female *S. frugiperda* [45,46]. The production of juvenile hormone inhibits the release of PBAN, resulting in decreased sex pheromone production in females and suppressing female receptivity, suggesting that mating significantly affects the release of pheromone compounds from glands by suppressing it, possibly as a mechanism to prevent attracting additional males after mating [47]. On the other hand, virgin *S. frugiperda* releases significantly higher quantities of pheromone compounds than mated females to secure mating first, suggesting that mating status directly modulates both the production and release of sex pheromones in *S. frugiperda* (Figure 1).

### 2.3. Mechanism of Pheromone Recognition by Male S. frugiperda

**Figure 2:**
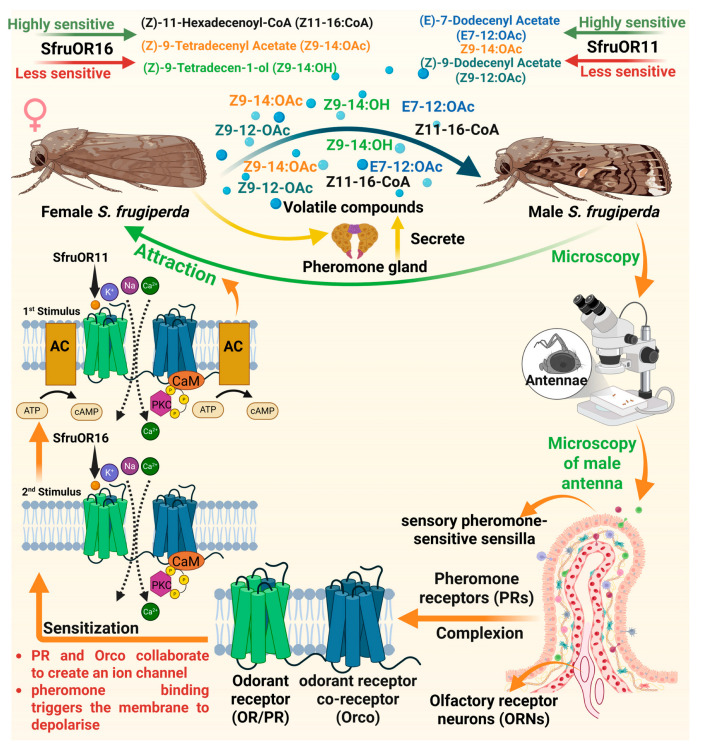
Mechanism of pheromone recognition by male *Spodoptera frugiperda*. The volatile compounds such as Z11–16:CoA, Z9–14:OAc, Z9–14:OH, E7–12:OAc, Z914:OAc and Z9–12:OAc emitted by female moths attract male moths. These are detected by pheromone receptors (PRs) on the antennae, where ion channels such as *SfruOR11* and *SfruOR16* trigger a dose-dependent response, sensitized by *Orco*, leading to membrane depolarization and behavioral attraction. “Figure created using Biorender.com”.

Pheromones consist of blend of several volatile compounds which are species-specific and secreted by the female *S. frugiperda* [48]. The female *S. frugiperda* produces Z11–16-OAc, Z9–12:OAc, Z9–14:OAc, Z9–14:OH and E7–12:OAc in their pheromone gland that are attractive to the male. These pheromone components are detected by the ORNs located in the sensory pheromone-sensitive sensilla, carried by the male antennae [49]. The ORNs detect pheromone components through a specific class of ORs known as pheromone receptors (PRs) in *S. frugiperda* [50]. Furthermore, ORs are seven-transmembrane receptors located on the dendrites of ORNs in *S. frugiperda*, and they function by forming complexes with a co-receptor called *Orco* [51,52]. PR and *Orco* collaborate to create an ion channel that opens when pheromones bind to it, triggering the membrane to depolarize [53,54]. Resultantly, the pheromone signal is transformed into an electrical signal that is transmitted to the brain for processing and integration, which then leads to the attraction behavior [48,49,50,51,52,53,54,55]. The PR, *Spodoptera frugiperda* odorant receptor 11 (*SfruOR11*), is highly sensitive to Z9–14:OAc and E7–12:OAc, which are the major sex pheromone components released by the female *S. frugiperda* [56]. Whereas less sensitive to Z9–12:OAc, which is the minor pheromone component [56]. *Spodoptera frugiperda* odorant receptor 16 (*SfruOR16*) responds weakly to pheromone components, Z9–14:OAc and Z11–16-CoA, but strongly to pheromone analog, Z9–14:OH. When these pheromone components bind to PRs, they trigger a large inward ion current, indicating strong receptor activation [56]. This receptor activation initiates the conversion of chemical signals into electrical signals, which are essential for accurate detection and the subsequent attraction behavior in male *S. frugiperda* (Figure 2).

#### Dose-Dependent Response of Male *S. frugiperda* to Sex Pheromones

The response of male *S. frugiperda* to sex pheromones is significantly influenced by the dose of pheromone components. In this context, Malo et al. [57] determined the antennal response of male *S. frugiperda* to female sex pheromone using electroantennography and observed varying antennal activity. The lower doses (0.01 µg) of sex pheromone components such as Z9–14:OAc and Z9, E12–14:OAc produce substantially weaker responses than the higher dose (10 µg), which produces the strongest response (Figure 2), indicating the optimal biosensing ability of male antennae. Furthermore, this differential responsiveness suggests a highly tuned olfactory system capable of detecting minute variations in pheromone concentration, presumably enabling males to locate conspecific females more precisely under varying signal intensity levels.

## 3. Mechanisms of CRISPR/CAS Editing in Insects

### 3.1. Basic Mechanism of CRISPR/Cas System

**Figure 3:**
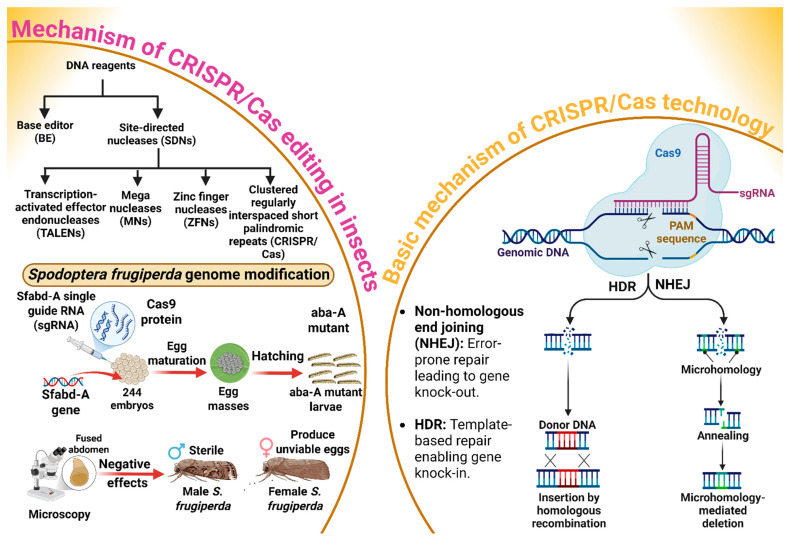
CRISPR/Cas9 gene editing in *Spodoptera frugiperda* involves using specific DNA reagents, such as base editors and site-directed nucleases, to modify the genome. The process focuses on editing the *Sfabd-A* gene, leading to the production of sterile male moths and unviable eggs. This mutation impacts the insect’s reproductive capabilities, which is key in pest control strategies. The CRISPR/Cas9 system operates through two primary DNA repair mechanisms: Non-homologous end joining (NHEJ) for gene knockouts, and homology-directed repair (HDR) for precise gene knockins. “Figure created using Biorender.com”.

CRISPR/Cas genome editing has evolutionary roots in the adaptive immune system of bacteria and archaea, where it functions as a defense mechanism against foreign genetic elements [58]. The basic mechanism of gene editing through the CRISPR/Cas system is explored in Figure 3. Base editors (BE) and site-directed nucleases (SDNs) are major proteins or DNA reagents that cause gene editing [59]. An SDN typically consists of a nuclease domain and the DNA-binding domain, which work together to create targeted breaks in DNA [58]. The DNA-binding domain identifies and binds to a specific DNA sequence, while the nuclease domain, often from the Fokl enzyme, cuts the DNA at that site [58]. Furthermore, transcription-activated effector endonucleases (TALENs), mega nucleases (MNs), zinc finger nucleases (ZFNs), and CRISPR/Cas are the four main types of SDNs used for gene editing [60]. The CRISPR/Cas9 genome-editing system relies on two components: the Cas9 protein, an endonuclease that introduces precise double-stranded breaks in DNA, and synthetic (sgRNA), which is engineered to direct Cas9 to specific sites or loci [61]. Two major pathways are used in this process. One is non-homologous end joining (NHEJ), which introduces the insertion or deletion of a targeted gene at the cleavage site, potentially disrupting the coding sequence of the targeted gene and leading to gene knock-out. Conversely, homology-directed repair (HDR) offers a precise alternative by utilizing a homologous DNA template to repair the break, thereby enabling targeted insertion called knock-in [61]. An extension of this technology is Co-CRISPR/Cas, in which multiple sgRNAs are required concurrently to target several loci within the genome. This multiplexed approach not only increases the throughput of gene editing but also accelerates the functional annotation of genes by facilitating simultaneous edits [62,63]. In this context, Wu et al. [64] explored the possibility of using the CRISPR/Cas9 system to modify the genome of *S. frugiperda* through the procedure mentioned in Figure 3. The results indicated that embryos injected with the *Spodoptera frugiperda* abdominal-A homeotic (Sfabd-A) sgRNA developed into larvae that displayed typical aba-A mutant phenotypes, including fused segments. Surprisingly, besides immatures, the adult *S. frugiperda* were also prone to the negative effects of mutation, so that males were sterile and females produced unviable eggs. These results suggest that the *S. frugiperda* genome can be effectively modified through the CRISPR/Cas system, demonstrating its potential as a powerful tool for inducing precise mutations (Figure 3). The successful generation of aba-A mutant phenotypes in larvae, coupled with the observed detrimental effects on adult fertility, highlights the system’s significant promise for advancing functional genomics. Moving forward, research should prioritize broadening the application of CRISPR/Cas9 across a wider array of insect species, particularly by adopting multiplexed CRISPR strategies, such as Co-CRISPR, to accelerate the functional annotation of genomes. Moreover, optimizing the system’s efficiency and precision while thoroughly investigating the long-term consequences of induced mutations will be crucial for fully realizing the potential of CRISPR-based technologies for agricultural innovation and pest control.

### 3.2. Application in Insects

CRISPR/Cas system has modified the genetic engineering process in biotechnology in various organisms and established genetically modified animal and cell models of many human diseases [65]. This technology provides high precision, versatility and efficiency in genome editing for functional genomics and genetic engineering in insects. This tool can alter the function of heritable genes by targeting the domains of the white gene (*Ex2Ex5*) in spotted wing *Drosophila*, *Drosophila suzukii* (Matsumura), resulting in disruption of pigmentation and visible phenotypic changes, especially in eye color [66]. Moreover, another study conducted by Lamb et al. [67] revealed that gene editing in *D. suzukii* can cause alterations in various characteristics, including body pigmentation, eye color, and wing fluorescence. Besides providing insights into gene function, CRISPR/Cas technology is also helpful for precisely editing the DNA of insect pests to suppress their populations or for developing novel insect genetic control strategies that cause developmental abnormalities [68]. Structural abnormalities in abdominal segmentations of tobacco cutworm, *Spodoptera litura*, Fabricius, were observed when manipulating the function of *Slabd-A*, a homolog of the abdominal-A gene [69]. Moreover, Zuo et al. [70] used the CRISPR/Cas9 as a tool to functionally validate resistance mutations in Beet armyworm, *Spodopera exigua*, Hubner by introducing a specific mutation in the ryanodine receptor gene, and this mutation confers high resistance to diamide insecticides, specifically chlorantraniliprole, cyantraniliprole and flubendiamide, demonstrating how the CRISPR/Cas9 system has revolutionized genetic engineering in insects by enabling precise modifications that not only advance our understanding of gene function but also hold promise for developing novel insect pest control strategies.

## 4. CRISPR/Cas-Mediated Disruption of the Olfactory System in *Spodoptera frugiperda*

### 4.1. Identification and Function of Key Olfactory Genes in Spodoptera frugiperda

**Figure 4:**
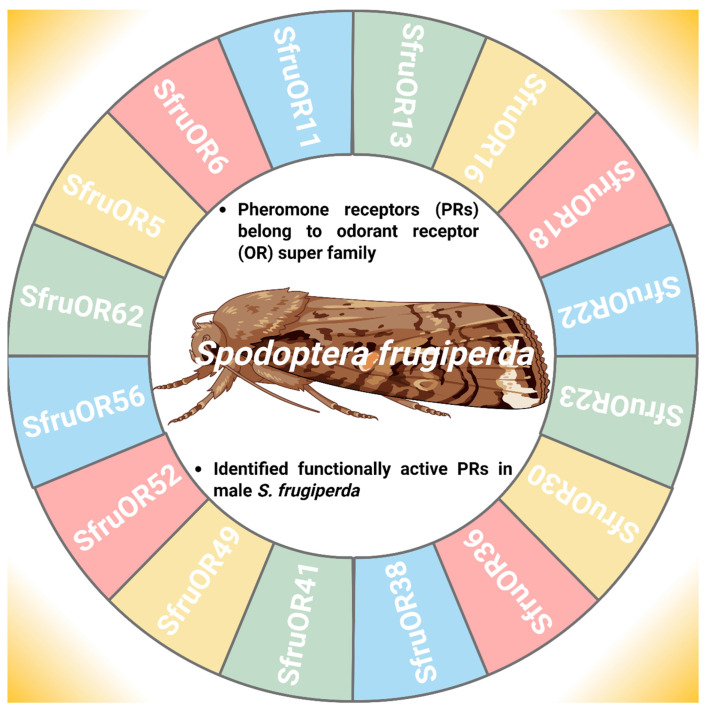
Odorant receptor genes involved in pheromone detection in *Spodoptera frugiperda*. “Figure created using Biorender.com”.

PRs refer to the members of the OR superfamily that facilitate intraspecific sexual communication between males and females, *S. frugiperda* [71]. The identification of functionally active PRs provides a key step in mate location and reproductive success (Figure 4) [72]. In this context, Guo et al. [56] functionally characterize the PRs in male antennae of *S. frugiperda* and found six candidate PRs (*Spodoptera frugiperda* odorant receptor 6 (*SfruOR6*), *SfruOR11*, *Spodoptera frugiperda* odorant receptor 13 (*SfruOR13*), *SfruOR16*, *Spodoptera frugiperda* odorant receptor 56 (*SfruOR56*), and *Spodoptera frugiperda* odorant receptor 62 (*SfruOR62*). Furthermore, Zhang et al. [71] first identified novel ORs (*Spodoptera frugiperda* odorant receptor 5 (*SfruOR5*), *Spodoptera frugiperda* odorant receptor 18 (*SfruOR18*), *Spodoptera frugiperda* odorant receptor 22 (*SfruOR22*), *Spodoptera frugiperda* odorant receptor 23 (*SfruOR23*), *Spodoptera frugiperda* odorant receptor 30 (*SfruOR30*), *Spodoptera frugiperda* odorant receptor 36 (*SfruOR36*), *Spodoptera frugiperda* odorant receptor 38 (*SfruOR38*), *Spodoptera frugiperda* odorant receptor 41 (*SfruOR41*), *Spodoptera frugiperda* odorant receptor 49 (*SfruOR49*), and *Spodoptera frugiperda* odorant receptor 52 (*SfruOR52*) responding to the sex pheromones in *S. frugiperda* because PRs fall into a single monophyletic clade in the OR family. These PRs are not exclusively found in male insect antennae; while they are more abundant and specialized in males, females also possess PRs for autodetection of their pheromones [73]. However, the expression of PR genes varies depending on the gender of the insect in such a way that expression of *SfruOR6*, *SfruOR11*, *SfruOR13*, *SfruOR16*, *SfruOR56*, and *SfruOR62* genes was higher in male antennae than female antennae because male antennae have more sensilla than female antennae [74,75]. The functional characterization of sex-biased PRs in *S. frugiperda*, with predominant expression in male antennae, provides promising targets for blocking the signal and its perception, thereby enhancing the efficiency of genetic pest management.

The ionotropic receptors (IRs) are also the most important family of insect olfactory receptors, which are widely expressed in both olfactory organs of insects [76]. IRs are derived from ionotropic glutamate receptors (iGluRs), are composed of 600–1000 amino acids, and have an architectural arrangement of two extracellular ligand-binding domains and three transmembrane domains [76]. In *S. frugiperda*, Sun et al. [77] identified expression of 10 IRs across different anatomical structures in male and female individuals, involved in sensory and reproductive functions. These IRs includes *Spodoptera frugiperda* ionotropic receptor 25a (*SfruIR25a*), *Spodoptera frugiperda* ionotropic receptor 60a (*SfruIR60a*), *Spodoptera frugiperda* ionotropic receptor 75d (*SfruIR75d*), *Spodoptera frugiperda* ionotropic receptor 64a (*SfruIR64a*), *Spodoptera frugiperda* ionotropic receptor 40a (*SfruIR40a*), *Spodoptera frugiperda* ionotropic receptor 100 (*SfruIR100*), *Spodoptera frugiperda* ionotropic receptor 41a (*SfruIR41a*), *Spodoptera frugiperda* ionotropic receptor 75p (*SfruIR75p*), *Spodoptera frugiperda* ionotropic receptor 76b (*SfruIR76b*), *Spodoptera frugiperda* ionotropic receptor 75q.1 (*SfruIR75q.1*). Furthermore, all these IRs majorly contribute to olfaction due to the expression of these IRs (except *SfruIR60a*) in male and female antennae rather than male and female proboscises, male and female tarsi, and female pheromone gland ovipositor. The gustatory receptors (GRs) are also the most important family of insect olfactory receptors, crucial for the peripheral coding of non-volatile compounds and essential for multiple behaviors including feeding [78]. For feeding *S. frugiperda* adults taste food by utilizing the GRs *Spodoptera frugiperda* gustatory receptor 1 (*SfruGR1*) and *Spodoptera frugiperda* ionotropic receptor 64a (*SfruGR2*), which are mainly expressed in male and female maxillae. In contrast, the *Spodoptera frugiperda* ionotropic receptor 64a (*SfruGR3*) and *Spodoptera frugiperda* ionotropic receptor 64a (*SfruGR9*) are involved in larval gustation [79]. The general odorant-binding proteins (GOBPs) are another class of olfactory proteins that serve as the link between external odorant molecules and ORs [80]. The insect GOBPs are small in size, with 15–17 kDa soluble proteins and concentrated in the lymph of chemosensory sensilla [81]. In *S. frugiperda*, the GOBPs are expressed at higher levels in adults than in other developmental stages and bind general plant volatiles and insecticides, ultimately providing a basis for controlling this insect pest. This knowledge can help explore plant volatile-sensing mechanisms and improve resistance to insecticides [82]. Furthermore, the *Spodoptera frugiperda* general odorant-binding protein 1 (*SfruGOBP1*) exhibits a lower binding affinity than *Spodoptera frugiperda* general odorant-binding protein 2 (*SfruGOBP2*), because the former binds to 4 of 38 general tested plant volatiles and 3 of 7 insecticides. In contrast, the latter binds to 21 volatiles and 4 insecticides. *SfruGOBP2* has more amino acid residues (E28, E31, E42, H7, S56, S79) compared to *SfruGOBP1* (F33, S56, and S65) showed a potentially strong binding affinity [82]. Besides the role of GOBPs in olfaction, the non-sensory OBP, odorant-binding protein 27 (OBP27), is highly expressed in the *S. frugiperda* fat body and mediates the transport of lipid metabolites during the eclosion process. Furthermore, it also supports the physiological function of *S. frugieda* due to its abundance in male reproductive organs, ultimately promoting mating [83]. Similarly to the OBPs, the chemosensory proteins (CSPs) are water-soluble, acidic proteins of about with a lenfht of 13–17kDa of approximately 120 to 170 amino acid residues [84,85], with an α-helical domain that forms a hydrophobic cavity [86]. In *S. frugiperda*, twenty-two *Spodoptera frugiperda* chemosensory proteins (*SfruCSPs*) with amino acid lengths between 107 and 233 and highly expressed in the larval or egg stage, indicating their functional complementation [87]. The larval inner endocuticle and outer epicuticle of *S. frugiperda* are also enriched with *Spodoptera frugiperda* chemosensory protein 1 (*SfruCSP1*) and *Spodoptera frugiperda* chemosensory protein 2 (*SfruCSP2*), which exhibited a high binding affinity with insecticides, chlorfenapyr, chlorpyrifos, and indoxacarb. Therefore, knocking down these proteins will significantly enhance the penetration of insecticides and increase *S. frugiperda*’s susceptibility to them [88].

### 4.2. Practice and Effects of CRISPR/Cas9-Mediated Orco Knockout

**Figure 5:**
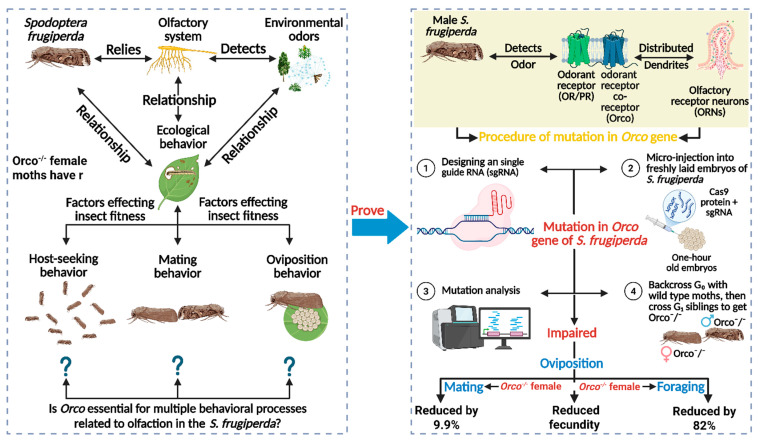
The role of the olfactory receptor co-receptor (*Orco*) gene in the olfactory system of *Spodoptera frugiperda* and its impact on behavioral processes, including host-seeking, mating, and oviposition. The mutation of the *Orco* gene using CRISPR technology impairs these behaviors, with significant reductions in mating, fecundity, and foraging efficiency. “Figure created using Biorender.com”.

The *S. frugiperda* relies exclusively on its olfactory system to detect the environmental odors, necessary for its ecological behavior, such as host-seeking, mating, and egg laying [89,90,91]. For the detection of odors from the environment, ORs in the olfactory system play a significant role, which are distributed on the dendrites of ORNs [92]. The *Orco* is an obligatory component that is required for dimerization with ORs through an intracellular loop to form a ligand-gated ion channel, which together is activated by odor molecules [93,94]. For mutating this *Orco* gene, Sun et al. [95] applied the CRISPR/Cas9 genome editing tool by designing an sgRNA targeting exon 2 using the ZiFit Tool. After combining the synthesized sgRNA with Cas9 protein, they were microinjected into freshly laid one-hour-old embryos of *S. frugiperda*. Then, injected embryos were incubated for hatching, and genomic DNA from larvae was screened for mutations using PCR and sequencing. A 4 bp deletion was identified, causing a frameshift and an early stop codon, resulting in a non-functional *Orco* protein. Mutant lines were established by backcrossing G_0_ individuals with wild-type moths, followed by screening G_1_ and developing homozygous *Orco^−/−^* mutants through G_1_ sibling crosses. *Orco* gene was successfully knocked out, confirmed by quantitative polymerase chain reaction (qPCR), showing near-absent *Orco* mRNA expression in mutants [95]. Resultantly, the physiological behavior, such as mating, oviposition and foraging behaviors of *S. frugiperda* was significantly impaired in such a way that the response of *Orco^−/−^* male moths to two sex pheromones, Z9–14: Ac and Z7–12: Ac, were abolished and therefore, *Orco^−/−^* male and female moths reduced the mating frequency by 100% as compared to wild type. Furthermore, *Orco^−/−^* female moths have reduced fecundity on the maize host plant as compared to wildtype, as well as *Orco^−/−^* larvae locate the food source 12.3 min longer than wild type larvae (2.2 min). Overall, the mutation in *Orco* causes impaired behavior in both larval and adult *S. frugiperda* (Figure 5), offering a precise, eco-friendly pest control strategy and reducing reliance on conventional insecticides.

## 5. Effects of *Orco* Knockout on Host Plant Volatile Perception in *Spodoptera frugiperda*

Insect pests use variety of plant volatiles to locate hosts for phytophagy and reproduction [96,97]. The perception of plant volatiles in *S. frugiperda* depends on ORs, with *Orco* being essential for the formation of functional ion channels in the membrane of ORNs located in the middle receptors of the male antennae [98,99,100]. The ORNa and ORNb in the male antennae of *S. frugiperda* females utilized specific maize volatiles, p-xylene and (+)-camphor, to attract the maize plant for oviposition and phytophagy [101]. However, these findings provide a foundation for sustainable pest management by introducing odor-based traps that effectively control *S. frugiperda* in maize fields. In addition, silencing the *Orco* gene in insects that perceive plant volatile odors is a promising insect control strategy [100,101,102]. To utilize this tool, Song et al. [103] knocked out the *Orco* gene in *S. frugiperda* using the CRISPR/Cas9 gene-editing system. It impaired the ability of *Orco* knock-out *S. frugiperda* to perceive plant volatiles, including benzaldehyde, β-pinene, cis-2-hexen-1-ol, jasmone, myrcene, and salicylaldehyde, suggesting that *Orco* is an excellent target for disrupting *S. frugiperda*’s normal behavior and providing a feasible pest control approach [103].

The *Orco* gene constitutes a 1422 bp coding sequence and both 5′ and 3′ untranslated regions, which play a critical role in gene expression. Furthermore, this gene encodes a protein of 473 amino acids, with a molecular weight of 5.3 kDa [95]. The CRISPR/Cas9 system specifically alters the 20 bp target sequence in exon 3 of the *Orco* gene, causing a 4 bp deletion that ultimately results in a frameshift and a truncated 72-amino acid protein, ultimately disrupting the formation of the functional Orco/OR heteromeric complex and impairing insect olfactory detection of plant volatiles [95].

## 6. Self-Limiting *Spodoptera frugiperda*: A Genetic Control Strategy

Until 2024, it was considered that achieving large-scale, effective CRISPR/Cas delivery systems for gene editing remained a critical bottleneck, since embryo microinjection is time-consuming, labor-intensive, and technically demanding, and is the main limitation making it unsuitable for field applications [104]. Furthermore, this approach has been successfully employed only in the laboratory, and large-scale field trials are necessary to validate the laboratory findings [63,105]. However, the introduction of self-limiting transgenic insects carrying lethal genes is the most promising genetic strategy for insect pest management [106]. In this context, a recent study by Reavey et al. [107] investigated the long-term effectiveness and practicality of releasing a genetically engineered, self-limiting strain of *S. frugiperda* (*OX5382G*) in a real-world agricultural environment in Brazil as a novel plant protection tool. To develop this strain, the embryos of *S. frugiperda* “Starkville” wild-type were injected with the *pOX5382* plasmid DNA and *piggyBac* mRNA encoding the transposase. The survivors were screened for the presence of the *DsRed2* marker to identify transgenic larvae (*OX5382G*). The *OX5382G* strain is self-limiting due to its female-specific mortality, which was conditional on the presence of deoxycycline in the larval diet. Therefore, males are released in the field, and this self-limiting factor reduces the female population [108]. After the release of laboratory-edited male moths into a maize field in Brazil, they showed no significant fitness penalties compared with wild-type males. They were able to disperse and mate effectively with wild females, and their survival rates were similar to those of their wild counterparts in terms of movement and mating ability. Furthermore, the *OX5382G* males possess a self-limiting gene that, upon mating with wild-type female moths in the field, causes the female offspring to die prematurely, thereby preventing their survival and reproduction [107]. Therefore, genetically modified insect release is emerging as a promising tool to suppress insect pest populations without contaminating the environment with insecticides [109,110,111].

The persistence of laboratory-edited insects in the field is a crucial factor in the success of field release. The lower persistence of the genetically modified strain of *S. frugiperda* (*OX5382G*) was observed, with no mutant moths detected in the corn field 25 days post-release, indicating that its self-limiting trait led to population collapse (die-off) shortly after release, preventing long-term persistence [107]. Because in a self-limiting strategy, the genetic modification is programmed to disappear from the population after several generations. However, a self-sustaining strategy is utilized to design a genetic element for enhancing frequency over generations, even when associated with a fitness cost, and seeded at a low proportion in the target population [112]. Regarding biosafety concerns, Reavey et al. [107] also stated that *OX5382G S. frugiperda* poses no significant risk to the environment or to human or animal health. Because the inserted proteins, such as tetracycline repressor protein (tTAV) and discosoma red fluorescent protein 2 (*DsRed2*), are non-toxic, non-allergenic, and do not adversely affect predators or parasitoids, the Comissão Técnica Nacional de Biossegurança (CTNBio) approved the commercial deployment of this strain for operational use in the agricultural land in Brazil [107]. Furthermore, to achieve an overflooding ratio (*OX5382G* males to wild males) in a 200-acre area, release rates of 27–200 males per acre per week were required. When considering multiple release points, this rate was reduced to 14–38 males per acre per week, accounting for incoming *OX5382G* males from neighboring areas, which is more practical for large-scale corn fields in Brazil [107]. More research is needed to develop this new approach for sustainable crop protection and resistance management across different countries and to assess its potential in the field. However, the large-scale release of mutant insects presents significant cost challenges, including high production costs, logistical complexities, and the need for continuous monitoring [107].

## 7. Practical Advantages and Limitations of CRISPR/Cas9

The CRISPR/Cas system achieves remarkable precision in targeting DNA by using sgRNAs to cleave DNA at specific locations, resulting in highly accurate genetic modifications [113]. First, it was applied in *D. melanogaster* and then used in silkworms, mosquitoes, moths, and other insect species [114]. Currently, this tool is widely used and highly effective in manipulating many biological functions of *S. frugiperda*, including growth and development, reproduction, sex determination, communication, and physiological functions [11,63,115,116,117,118,119]. Moreover, this technology is lower-cost, user-friendly, straightforward, and simplifies the editing process compared to more advanced gene-editing tools [119]. In genome editing, Cas enzymes, including CRISPR-associated protein 9 (Cas9), CRISPR-associated protein 12a (Cas12a), CRISPR-associated protein 12b (Cas12b), CRISPR-associated protein 13 (Cas13), and CRISPR-associated protein 7–11 (Cas7–11), are widely used and offer distinct advantages [120]. Cas12a and Cas12b identify T-rich protospacer adjacent motif (PAM) sites (TTTVs), which increase target-site flexibility and genome-editing effectiveness [121], whereas Cas13 cleaves RNA rather than DNA and does not require a PAM site [122]. Generally, Cas enzymes other than Cas9 generate fewer off-target effects, exhibit lower cytotoxicity, and are smaller, thereby facilitating a more efficient and effective delivery system [120]. For enhancing the efficiency of CRISPR/Cas9 in *S. frugiperda*, the strong tissue-specific active promoters are required for effective expression of Cas9 and sgRNAs, such as *Spodoptera frugiperda* heatshock protein 20.15 (*SfHsp20.15*), *Spodoptera frugiperda* heatshock protein 20.71 (*SfHsp20.71*), *Spodoptera frugiperda* heatshock protein 20D (*SfHsp20D*), *Spodoptera frugiperda* heatshock protein 70D (*SfHsp70D*) from *S. frugiperda* have been identified and characterized [123]. Regarding the limitations of this system, off-target effects are the most common challenge in CRISPR/Cas9 applications, as the Cas9 enzyme can cleave DNA at unintended locations [124]. For mitigating this issue, the case enzyme variants, including *Streptococcus pyogenes* Cas9-High fidelity variant 1 (*SpCas9-HF1*) and *xCas9*, have high specificity and edit target gene sequences precisely without introducing mutations at off-target sites [125]. Microinjection is another significant limitation on the efficiency of the CRISPR/Cas9 delivery system, due to its time-consuming, labor-intensive, and technically demanding nature, as well as its confinement to the laboratory [104]. To address this issue, a revolutionary technique, nanoparticle-mediated CRISPR/Cas9 delivery, for accurate gene editing of *Spodoptera frugiperda* 9 (Sf9) cell lines, is crucial for the long-term success of this technology-based insect pest control [126,127].

## 8. Conclusions and Future Perspectives

Volatile compounds play a crucial role in sexual communication between conspecific *S. frugiperda*, and their emission is highly age- and mating-status-dependent. In male *S. frugiperda*, the olfactory system is diversified due to containing olfactory receptor families, including PR, IRs, GRs, GOBPs, and CSPs, which play a significant functional role, including mating, feeding, and providing resistance. Among these receptor families, *Orco* is the obligatory component of ORs, is essential for OR localization to dendritic membranes, and thus is necessary for odorant detection. Concerning the importance of *Orco* in the olfactory system, a CRISPR/Cas9-based genetic control strategy was employed to knock out the *Orco* gene in *S. frugiperda*, ultimately impairing ecological behaviors. Despite its limitations, this CRISPR/Cas9 system also has advantages and should be explored for large-scale mutagenesis. A novel self-limiting genetic control strategy for *S. frugiperda* has been practically investigated at a large scale in Brazil by introducing transgenic *S. frugiperda* with no significant environmental risk. Looking ahead, the behavioral responses of *S. frugiperda* mutants generated using CRIAPR/Cas9 and self-limitation techniques should be investigated across multiple countries.

## Data Availability

No new data were created or analyzed in this study. Data sharing is not applicable to this article.

## Abbreviations

AAT	Alcohol acetyltransferase
ACC	Acetyl-CoA carboxylase
BE	Base editors
C16:0-CoA	Saturated 16-carbon fatty acyl-CoA
Cas12a	CRISPR-associated protein 12a
Cas12b	CRISPR-associated protein 12b
Cas13	CRISPR-associated protein 13
Cas7–11	CRISPR-associated protein 7–11
Cas9	CRISPR-associated protein 9
CRISPR-Cas9	Clustered Regularly Interspaced Short Palindromic Repeats
CTNBio	Comissão Técnica Nacional de Biossegurança
CTNBio	Comissão Técnica Nacional de Biossegurança
DNA	Deoxyribonucleic acid
DsRed2	Discosoma red fluorescent protein 2
E7–12:OAc	(E)-7-dodecenyl acetate
FARs	Fatty acyl CoA reductases
FAS	Fatty acid synthase
HDR	Homology-directed repair
MAG	Male’s accessory gland
MNs	Meganucleases
mRNA	Messenger RNA
NHEJ	Non-homologous end joining
Orco	Odorant receptor co-receptor
ORNs	Olfactory receptor neurons
ORs	Odorant receptors
PAM	Protospacer adjacent motif
PBAN	Pheromone biosynthesis-activating neuropeptide
PRs	Pheromone receptors
qPCR	Quantitative polymerase chain reaction
RNA	Ribonucleic acid
SDNs	Site-directed nucleases
Sfabd-A	*Spodoptera frugiperda* abdominal-A homeotic
SfHsp20.15	*Spodoptera frugiperda* heatshock protein 20.15
SfHsp20.71	*Spodoptera frugiperda* heatshock protein 20.71
SfHsp20D	*Spodoptera frugiperda* heatshock protein 20D
SfHsp70D	*Spodoptera frugiperda* heatshock protein 70D
SfruOR11	*Spodoptera frugiperda* odorant receptor 11
SfruOR13	*Spodoptera frugiperda* odorant receptor 13
SfruOR16	*Spodoptera frugiperda* odorant receptor 16
SfruOR18	*Spodoptera frugiperda* odorant receptor 18
SfruOR22	*Spodoptera frugiperda* odorant receptor 22
SfruOR23	*Spodoptera frugiperda* odorant receptor 23
SfruOR30	*Spodoptera frugiperda* odorant receptor 30
SfruOR36	*Spodoptera frugiperda* odorant receptor 36
SfruOR38	*Spodoptera frugiperda* odorant receptor 38
SfruOR41	*Spodoptera frugiperda* odorant receptor 41
SfruOR49	*Spodoptera frugiperda* odorant receptor 49
SfruOR5	*Spodoptera frugiperda* odorant receptor 5
SfruOR52	*Spodoptera frugiperda* odorant receptor 52
SfruOR56	*Spodoptera frugiperda* odorant receptor 56
SfruOR6	*Spodoptera frugiperda* odorant receptor 6
SfruOR62	*Spodoptera frugiperda* odorant receptor 62
sgRNA	Single-guide RNA
SpCas9-HF1	Streptococcus pyogenes Cas9-High-fidelity variant 1
TALENs	Transcription activator-like effector nucleases
tTAV	Tetracycline repressor protein
Z11–16-CoA	(Z)-11-hexadecenoyl-CoA
Z7–12:CoA	(Z)-7-dodecenyl acetate
Z7–12:OAc	(Z)-7-dodecenyl acetate
Z9–12:OAc	(Z)-9-dodecenyl acetate
Z9–14:OAc	(Z)-9-tetradecenyl acetate
Z9–14:OAc	(Z)-9-tetradecenyl acetate
ZFNs	Zinc finger nucleases
